# Effect of Surgical Mask use on Peak Physical Performance During Exercise Treadmill Testing-A Real World, Crossover Study

**DOI:** 10.3389/fphys.2022.913974

**Published:** 2022-05-24

**Authors:** Mayank Dalakoti, Cheryl Long, Arshia Bains, Andie Djohan, Isabel Ahmad, Siew Pang Chan, Jieli Kua, Po Fun Chan, Tee Joo Yeo

**Affiliations:** ^1^ National University Heart Centre, Singapore, Singapore; ^2^ Yong Loo Lin School of Medicine, National University of Singapore, Singapore, Singapore

**Keywords:** exercise testing, physiological stress, COVID–19, surgical mask, environmental physiology

## Abstract

**Background:** Mask wearing is an essential strategy to combat the spread of SARS-CoV-2. Some individuals may wear masks during physical activity to reduce disease transmission. This study aimed to investigate the real-world effect of wearing a surgical face mask on physiological parameters at peak exercise in healthy individuals.

**Methods:** In this crossover design study, participants underwent maximal treadmill electrocardiogram exercise tests using the Bruce protocol on two separate occasions, once with a standard 3-ply surgical face mask and once without. Heart rate, oxygen saturation, blood pressure, rate pressure product, metabolic equivalents (METS) and total exercise time were measured. Subjective rate of perceived exertion was also assessed using the modified Borg Scale.

**Results:** 50 adults (mean age = 31.7 ± 6.5 years; 27 males) completed both treadmill tests. Mask wearing resulted in a significant reduction in peak METS by 1.5 units, maximum speed by 0.5 km/h, exercise time by 68.4 s with a significantly lower peak heart rate by 4.4 bpm, and lower percentage of age-predicted maximum heart rate by 2.5% (*p* < 0.001 for all parameters). During each corresponding stage of the Bruce protocol, the average modified Borg score was found to be significantly higher in subjects exercising with mask after adjusting for age, gender and body mass index (*p* < 0.03).

**Conclusion:** In a cohort of healthy individuals, wearing of a surgical face mask during maximal treadmill exercise lead to reduced physical performance and increased rate of perceived exertion. Individuals exercising with surgical masks need to be mindful of these limitations while undergoing physical training in order to differentiate these physiological responses from symptoms of early respiratory illness.

## Introduction

The SARS-CoV-2 pandemic has proven to be a long battle, with new emerging variants such as Omicron found to be increasingly infectious. Almost 2 years into the global pandemic, infection control measures such as mask wearing, physical distancing and regular hand washing have been instrumental in helping to control and reduce the rate of viral transmission, and have been recommended by the World Health Organisation (WHO) (Chu et al., 2020; CDC, 2022). Even as more countries begin to open their borders and transition from a pandemic to endemic phase, these safety measures are likely to remain in place for the foreseeable future.

Beginning 7 April 2020, the Singapore government implemented stringent infection control measures as part of a nation-wide circuit breaker to break the chain of COVID-19 transmission. Specifically, wearing of masks when leaving the house became mandatory by law for all individuals 6 years old and above, with the exception of those engaging in strenuous exercises (MOH, 2020). The WHO has also advised against wearing masks during vigorous physical activity based on the potential detrimental effects of carbon dioxide accumulation during the wearing of face masks (Shurlock et al., 2021).

Certain individuals choose to keep their masks on during physical activity, such as those at high risk of complications from COVID-19 with underlying cardiopulmonary conditions (CNA, 2021). Others may also voluntarily wear their masks during exercise to minimize the risk of contracting COVID-19.

Since the start of the pandemic, several studies have investigated the effect of mask wearing on physiological response during exercise in healthy individuals. Studies by Driver et al. (2022) and Fikenzer et al. (2020) showed a significant impairment in physical performance with the use of a face mask. Other studies by Shaw et al. (2020) and Epstein et al. (2021) showed no significant impact of face mask use on physical performance. Alongside the conflicting data, there were also key differences in study conduct, with earlier studies employing the use of a face mask worn together with a spirometry mask. Poon et al. (2021) conducted a crossover trial of 13 individuals that found no significant differences in physiological outcomes during exercise with the use of a face mask, although an increased rate of perceived exertion was reported with mask use on vigorous exertion. Fischer et al. (2022), conducted a similar trial in a population including those with coronary artery disease or hypertension, and concluded that face mask use during exercise reduced peak power output without differences in hemodynamic parameters.

We hypothesised that wearing of surgical mask during exercise would lead to reduced peak physical performance, with increased rate of perceived exertion compared to exercising without a mask. Therefore, the present study was designed to investigate the real-world effect of wearing surgical masks on peak physical performance during maximal exercise in a cohort of healthy individuals.

## Materials and Methods

### Study Design

This was a crossover study where each subject served as his or her own control. The order of masked and unmasked treadmill test was randomly assigned based on study site. Those at National University Hospital (NUH) would start with a mask, those in Ng Teng Fong General Hospital (NTFGH) would start without a mask.

### Participants

The study population consisted of healthcare workers from NUH and NTFGH in Singapore.

Participants included were aged between 21 and 60 years old. Exclusion criteria were: pregnancy, implanted permanent pacemaker, consumption of any medications affecting heart rate, any known cardiac pathology, symptoms during exercise suggestive of underlying cardiorespiratory disease, physical impairment limiting treadmill performance or uncontrolled hypertension (systolic >160 mmHg or diastolic >100 mmHg).

Informed consent was obtained from all participants prior to the test. The study was approved by the local institutional review board.

Demographic data was collected via a questionnaire prior to exercise. Baseline physiological data such as blood pressure (BP), heart rate (HR), height and weight, as well as past medical history, medications and any recent illness were recorded.

### Treadmill Exercise Electrocardiogram (TMX) Test

All TMX tests were performed in a standardized manner using the Bruce protocol on an exercise treadmill (General Electric Company, T-2100, Michigan, United States) in NUH or NTFGH (Bruce Protocol Stress Test, 2021). All subjects performed the TMX test to subjective exhaustion on two separate occasions, once while wearing a standard surgical 3-ply medical grade equivalent mask and once without. The test was concluded at the point of subjective exhaustion of participants. The second TMX test was scheduled within 1 week after the first test. For tests done with mask on, subjects were instructed to conform to the manufacturer’s instructions including covering the nose and ensuring a snug fit, and to keep their mask on for the duration of the TMX test including the recovery phase. Minor adjustments to the masks were allowed during the test, as long as both the nose and mouth were covered throughout. This was done to reflect real-world use of masks.

Both tests were conducted pre-meal and at similar time of day. Participants were advised to refrain from vigorous physical activity the day before the test, and to maintain adequate hydration. No further visits or follow-ups were required after completion of both TMX tests.

### Parameters

During the TMX test, data collected included heart rate (HR), oxygen saturation (SpO2), blood pressure (BP), rate pressure product (RPP), metabolic equivalents (METS) and total exercise time.

METS was calculated automatically by the treadmill algorithm, namely:METS = [(speed in miles per hour x 26.8 × 0.1) + (grade in percent/100 × 1.8 x speed x 26.8) + 3.5]/3.5.

Subjective rate of perceived exertion (RPE) was also assessed using the modified Borg Scale (score of 0–10). This was assessed every 3 min for stage 1–3 of the Bruce protocol and every minute from stage 4 onwards (CDC, 2021).

Medical records were not assessed, and no biological samples were collected. All information collected was anonymized. Participants were informed of any abnormal findings from the investigations.

### Data Analysis

The data were presented as frequency (%) or mean ± standard deviation, depending on their nature. Exploratory data analyses were performed with paired *t*-test. In view of the repeated-measures, the confirmatory analyses concerning the differences in effects and occurrence of adverse events with and without mask during the TMX test were performed with the subject-specific random-intercept model. This is a mixed effect model that could account for the repeated measures. The likelihood ratio test was applied for model selection, with the default random-intercept model compared with the more general random-slope model. The 95% confidence intervals were reported for ascertaining statistical significance. Data were analysed with Stata MP version 17.0. All statistical tests were conducted at 5% level of significance.

### Statistical Power Calculation

The statistical power calculation was performed in the context of two-level subject-specific random-intercept model. The level of significance was fixed at 5%, while the variances of errors at both levels were considered for the above-mentioned models.

The computations confirmed that a statistical power of >85% was achieved for the estimated models given the effect sizes, sample size and number of observations nested in the groups (masked v unmasked). As such, the identified significant effects were not likely to be the result of chance.

## Results

### Baseline

The final sample size consisted of 50 individuals (27 male, 54.0%; mean age: 31.7 ± 6.5 years) who met the eligibility criteria and had successfully completed both TMX tests (Table 1). The majority had exercised regularly, and 1 (2%) had an underlying medical condition (hypertension). Regular exercise was defined as participation in any form of sports or exercise for at least 20 min per occasion, for three or more days a week (Ministry of Health, 2020).

**TABLE 1:** Baseline characteristics.

Variables	N (%)/Mean ± SD
Biodata	
Male gender	27 (54.0%)
Age (years)	31.7 ± 6.5
Height (cm)	165.9 ± 8.9
Weight (kg)	63.0 ± 10.1
Body Mass Index (kg/m2)	23.0 ± 3.1
Past Medical History	
Cardiorespiratory disease	0 (0.0%)
Hypertension	1 (2.0%)
Social History	
Smoking	0 (0.0%)
Regular Exercise	34 (68.0%)

### Exercise Parameters

Masked and unmasked individuals achieved 97.2% and 99.6% of age-predicted maximum heart rate, in keeping with maximum exertion. Subjects who were masked reported a significantly lower exercise time, peak METS, maximum speed and peak HR by 63.9 s, 1.5 units, 0.4 km/h and 4.6 bpm, respectively after adjusting for age, gender and body mass index (BMI).

Participants exercising without mask achieved 99.6 ± 5% of their age-predicted maximum heart rate, calculated using the Karvonen formula (Davis and Convertino, 1975), where maximum heart rate = 220—age. This was significantly reduced following wearing of surgical mask, by 2.5% (*p* < 0.001), after adjusting for age, gender and BMI.

Compared to unmasked tests, wearing of surgical masks led to increased heart rate recovery, lower peak systolic BP, lower peak SpO2, higher maximum RPP and higher peak modified Borg score, although these did not reach statistical significance (Table 2).

**TABLE 2:** Subject-specific random-intercept models^a^.

Outcomes	Masked	Unmasked	Coefficient (difference in masked vs. unmasked)	95% confidence intervals	*p*-value
Exercise time (sec)	814.0 ± 163.8	882.4 ± 184.5	−63.9	−86.8 to−41.0	<0.001
Peak METS	16.1 ± 3.1	17.6 ± 3.2	−1.5	−2.0 to −1.0	<0.001
Maximum speed (km/h)	7.8 ± 0.9	8.3 ± 0.9	−0.4	−0.6 to −0.3	<0.001
Peak HR (bpm)	183.0 ± 10.5	187.4 ± 9.9	−4.6	−6.6 to −2.5	<0.001
Percentage of age-predicted maximum heart rate (%)	97.2 ± 4.8	99.6 ± 5.0	−2.5	−3.5 to −1.4	<0.001
Peak SBP (mmHg)	182.3 ± 22.7	185.8 ± 24.8	−3.8	−10.1 to 2.5	0.235
Max rate pressure product	45998.2 ± 64846.1	30161.9 ± 5250.0	16001.8	−1961.2 to 33964.8	0.081
SpO2 (%) at peak exercise	92.1 ± 6.7	93.1 ± 5.8	−1.0	−3.1 to 1.1	0.356
Modified Borg score at peak exercise	7.5 ± 1.8	7.1 ± 2.2	0.4	−0.1 to 0.8	0.123
Heart rate recovery^b^(bpm)	28.9 ± 9.9	27.7 ± 8.6	1.4	−1.0 to 3.8	0.241

a adjusted for age, gender and BMI. (METS, metabolic equivalents; HR, heart rate; SBP, systolic blood pressure; SpO2, Oxygen saturation).

b Heart rate recovery defined as difference between heart rate at peak exercise and 1 min into recovery.

The two-level subject-specific random-intercept models, constructed with the repeated outcomes nested within the subjects, were found to be adequate. First, the random effects were significant. This suggests that the random-intercept models could explain the variations in the outcomes more satisfactorily than the models without hierarchical data structures. Next, the intraclass correlations were above 0.5 for the above-mentioned models. The random-slope models provided no significant added values to the random-intercept models. As such, the random-intercept models were chosen on parsimonious grounds.

### Subjective Rate of Perceived Exertion

**FIGURE 1:**
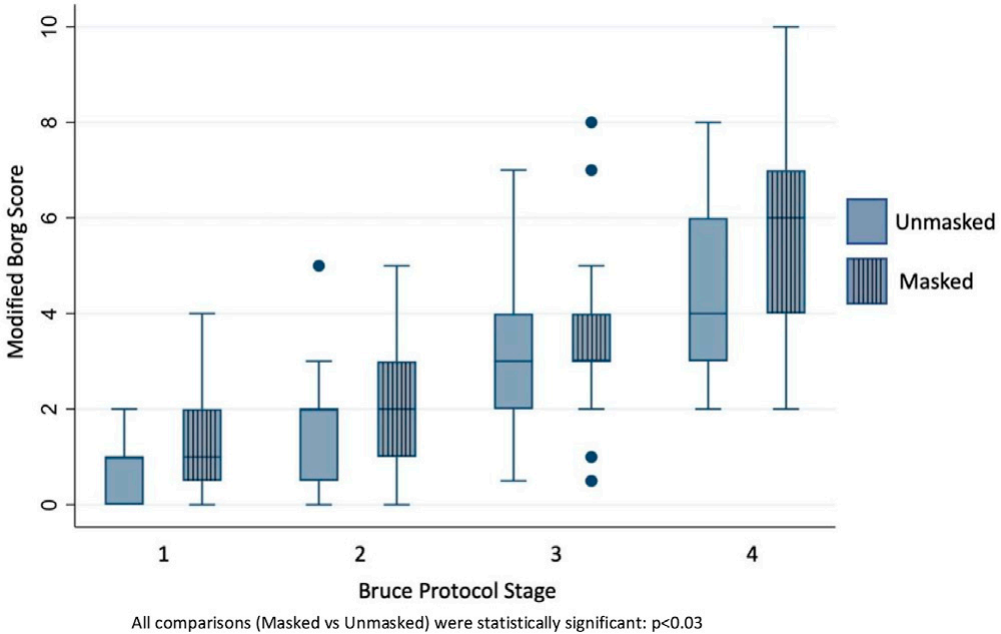
Modified Borg scores at respective Bruce stages of unmasked vs. masked exercise treadmill test.

**FIGURE 2:**
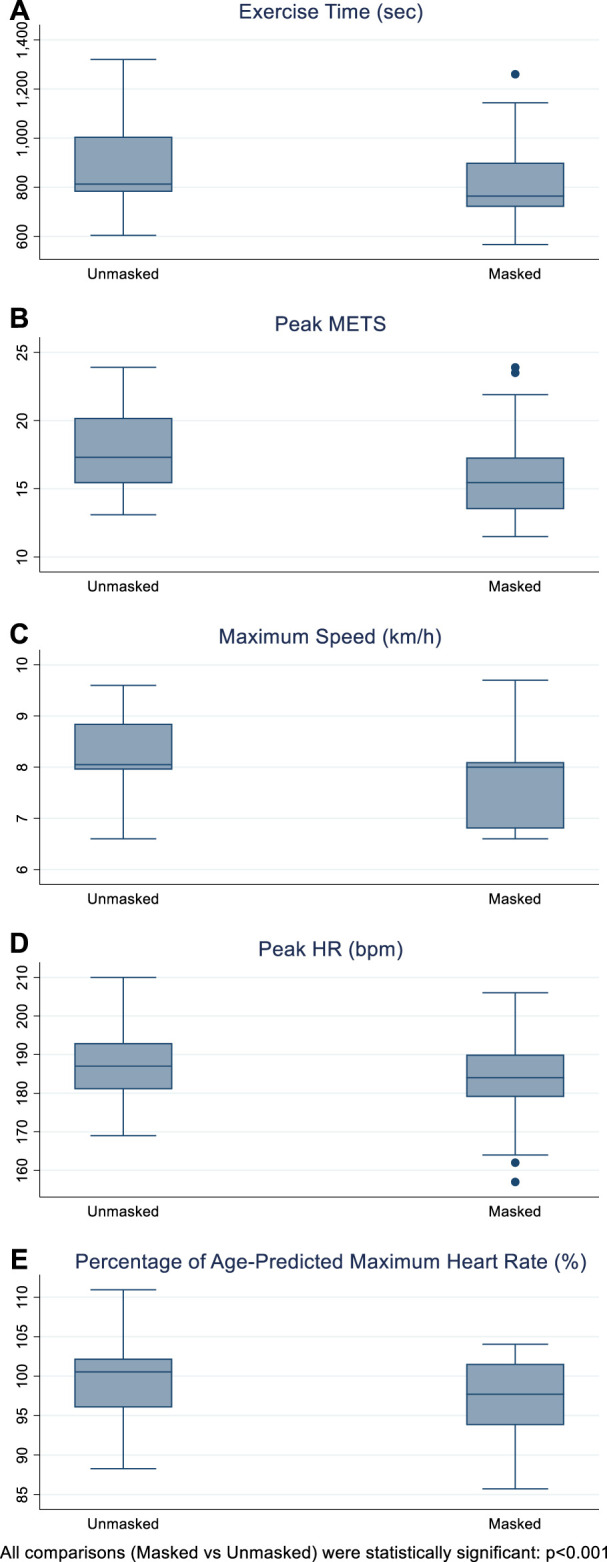
Exercise time (seconds), peak metabolic equivalents (METS), maximum speed (km/hour), peak heart rate (beats per minute), percentage of age-predicted maximum heart rate (%), in masked and unmasked individuals.

At each corresponding stage of the Bruce protocol, the average modified Borg score was found to be significantly higher in subjects exercising with a mask compared to those exercising without a mask, after adjusting with age, gender and BMI (Stage 1: 0.6 vs. 1.0, *p* = 0.001; stage 2: 1.6 vs. 2.1, *p* < 0.001; stage 3: 3.0 vs. 3.4, *p* = 0.029; stage 4: 4.6 vs. 5.8 *p* < 0.001, unmasked vs. masked respectively). (Figures 1, 2).

### Effect of Gender

The two-level subject-specific random-intercept models were constructed with age, gender and BMI as adjusting covariates.

Other things being equal, male subjects had a significantly higher average value for exercise time by 160.3 s (95% C.I.: 76.5–244.2), peak METS by 3.0 (95% C.I.: 1.5–4.5), maximum speed by 0.9 km/h (95% C.I.: 0.5–1.3) and peak SBP by 15.1 mmHg (95% C.I.: 4.9–25.3) compared to their female counterparts.

There was no significant gender effect identified for percentage peak HR, max rate pressure product, SpO2 (%) at peak exertion, modified Borg score at peak exertion and heart rate recovery (bpm).

However, the non-significant gender variable was kept in these models for completeness sake. Moreover, there would not be an impact on the estimated effect size of interest (masked vs. unmasked) given the sample size (*n* = 50 in 2 groups) and the number of covariates (4).

### Safety

Both masked and unmasked tests were comparable in safety. None of the participants reported any symptoms such as chest pain, syncope or giddiness, and no tests were stopped prematurely in either group. No significant ventricular arrhythmia occurred in either group. No significant ST segment changes were noted in the unmasked group. There was no significant difference between the masked and unmasked (46.0% vs. 46.8%; p: 0.882) tests in terms of occurrence of isolated ventricular ectopics.

There was 1 false positive TMX test in the masked group, in whom asymptomatic 2 mm horizontal ST segment depressions during stage 4 of the TMX test were noted. The participant was informed of the abnormal TMX test result and subsequently assessed with a myocardial perfusion imaging scan which revealed normal coronary perfusion with no ischemia.

## Discussion

In our crossover study of healthy individuals, we found that wearing a standard 3-ply surgical face mask while engaging in treadmill exercise reduced peak physical performance compared to that while unmasked. From a safety standpoint, exercise with mask use was generally safe in our cohort, with no significant adverse events. To date, this is the largest trial evaluating the use of a surgical mask alone in a real-world context, and helps to weigh in strongly on the debate of the impact of surgical mask use during exercise.

The peak heart rate and percentage of age-predicted maximum heart rate achieved by participants exercising during mask wearing was significantly lower. Even though peak heart rate achieved was lower in the masked group, the maximum rate pressure product between both groups was comparable, with a numerically higher value achieved in the masked group that was not statistically significant (45998.2 vs. 30161.9, *p* = 0.081). This observation suggests there was comparable maximal exertion in both groups of participants.

Overall, our findings suggest early physiological fatigue with mask wearing during exercise as the reason for earlier termination of the stress test.

This was consistent with an increased RPE observed in the group exercising with a mask. The RPE during exercise comprises the integration of cardiovascular, respiratory and musculoskeletal systems (Borg, 1982). We postulate that the combination of these factors-increased perceived dyspnoea and discomfort of mask usage during maximum exertion with a mask leads to an increased RPE and earlier stoppage due to fatigue, with resultant reduction in peak METS, speed and exercise time achieved.

To date, several studies have investigated the effect of mask wearing on physiological response during exercise with conflicting results (Refer to Annex) Our findings are in agreement with that by Driver et al. (2022) whereby use of a cloth mask during treadmill exercise with an additional cardiopulmonary exercise test (CPET) mask also led to a reduction in peak physical performance, with a reduction in exercise time and peak heart rate achieved. Although RPE at point of exhaustion was unaffected by mask use, the dyspnoea ratings were higher and the authors hypothesized that discomfort from mask wearing led to reduced exercise performance. This was also observed in studies by Fikenzer et al. (2020), Egger et al. (2022) and Poon et al. (2021).

Conversely, other studies such as those by Lässing et al. (2020), Umutlu et al. (2021), Shaw et al. (2020), Roberge et al. (2012) and Epstein et al. (2021) have shown no significant impact of masked exercise on RPE. In contrast to our findings, studies by Lassing, Umutlu and Roberge noted an increase in maximum heart rate with the use of a mask. There were differences in exercise protocol from our study, with Roberge et al. (2012) employing continuous treadmill exercise at 5.6 km/h for 1 h and Lässing et al. (2020) employing continuous cycle ergometry at participants’ highest oxidative metabolic rate. Although Umutlu et al. (2021) employed a similar incremental treadmill protocol as our study, the participants included were sedentary individuals, who may have a different response to exercise stress testing with a mask. Furthermore, the small sample sizes in these studies (ranging from 14 to 20 subjects) makes it difficult to generalize their findings.

Compared to other studies, our study has numerous strengths.

We assessed the use of a surgical mask in a real-world context. Most studies have previously used an additional spirometry mask worn over the face mask (Fischer et al., 2022). We would expect studies with double mask use to show early fatigue and reduced peak physical performance. Use of a surgical mask alone may enable more accurate characterisation of the real-world experience of exercise with a surgical mask.

Our study has one of the larger cohorts thus far with 50 participants. We employed a crossover study design, where each individual acted as his or her control. This would help to reduce confounding.

Our main modality of testing was the exercise treadmill. This modality of exercise stress was also used by Driver and Ahmadian (Ahmadian et al., 2021), while other studies by Epstein and Egger have used the cycle ergometry exercise test. Earlier studies have shown that exercise treadmill testing typically produces a higher heart rate response compared to cycle ergometry (Niederberger et al., 1974). In our study, we were able to show a reduction in peak physical performance with mask use via the exercise stress treadmill modality. These findings should also be applicable to individuals who choose to cycle for exercise.

## Limitations of the Study

Our study had several limitations. Firstly, our sample population mainly comprised of young, healthy and physically active adults. Hence, this data cannot be directly generalized to other populations such as the elderly and individuals with cardiopulmonary comorbidities. Secondly, we only used one type of standard surgical 3-ply medical grade equivalent mask for this study. Different types of mask models and designs may have different effects on physiological stress during exercise. Thirdly, the recuperation time between the two exercise treadmill tests was not standardized. This could influence the performance of the participants. However, the mean/median time between tests was 7/8 days, which provided sufficient time for recovery between bouts of exercise. Participants were also advised not to engage in strenuous physical activity the day before participating in the TMX test.

We did not utilise gas exchange for objective measurement of oxygen consumption and carbon dioxide production. However, our study aimed to mimic actual physical activity by requiring participants to wear only 1 face mask without any additional spirometry mask over that. In addition, we were unable to accurately measure respiratory rate during exercise as manual counting was not feasible. This would be an important surrogate measure of physiological stress. Finally, we did not employ the use of biochemical parameters such as lactate upon cessation of exercise, as a measure of metabolic stress.

## Conclusion

Our study in a cohort of healthy individuals showed that wearing surgical face masks significantly impacts physiological parameters at peak exercise, although it is generally safe. As the COVID-19 pandemic continues, individuals exercising with surgical masks need to be mindful of these limitations while undergoing physical training in order to differentiate the physiological responses of mask use from symptoms of early respiratory illness.

These findings can help inform individuals on their exercise practices, as well as broader public health policy decisions balancing safety of exercise and protection from respiratory illness. People choosing to exercise with a mask should work closely with a healthcare professional to do so in a safe manner, with their exercise prescription reviewed for frequency and intensity if required. Further studies in individuals with cardiopulmonary comorbidities are necessary to ascertain physiological impact and safety of masked exercise on these patients.

## Data Availability

The raw data supporting the conclusion of this article will be made available by the authors, without undue reservation.
