# Supplementary material for: Effect of Surgical Mask use on Peak Physical Performance During Exercise Treadmill Testing-A Real World, Crossover Study
Source: Front Physiol. 2022 May 24;13:913974. doi: 10.3389/fphys.2022.913974 (PMC9171022; doi:10.3389/fphys.2022.913974)
Supplement: Supplementary file 1 [file DataSheet2.docx]

**Annex**

| **Comparison of Research Papers** | | | | | |
| --- | --- | --- | --- | --- | --- |
| **Authors** | **Participant Profile** | **Exercise Test** | **Mask Worn** | **Variables Measured** | **Results** |
| Our paper | N = 50 recreationally active participants  (27 males, 23 females) | Treadmill ECG Exercise Test | Standard Surgical 3-ply Medical Grade Equivalent Mask | HR, SpO2, RR, BP, ECG, RPP, METs, Total exercise time  Subjective RPE using Borg Scale | Surgical mask wearing significantly impacted peak exercise performance, with significant reductions in peak METS, maximum speed, exercise time; lower peak heart rate and percentage of age-predicted maximum heart rate (p<0.001 for all parameters). RPE as assessed by modified Borg score was significantly higher at each corresponding level of Bruce protocol in masked individuals. |
| Ade (2021) et al [22] | N = 11 recreationally active participants  (5 males, 6 females) | Incremental ramp exercise tests to exhaustion on a cycle ergometer | Surgical mask (USA ASTM F2100)  N95 NIOSH approved 1570 respirator | HR, SpO2, RR, BP, O2, PO2, CO2, PCO2  Dyspnea and RPE | N95 and surgical masks did not impact maximal exercise capacity and there was no arterial hypoxemia, exercise intolerance and changes in cardiovascular variables during cycling exercise. However, ratings of dyspnea were significantly increased. |
| Ahmadian (2021) et al [20] | N = 144 recreationally active participants  (72 males, 72 females) | Submaximal and maximal exercises using Bruce treadmill protocol | N95 mask  Surgical mask | SBP, HR, Presence of chest pain, ECG changes, RPP, SpO2  venous blood sample pre and post exercise (RBC, WBC, Neutrophils, haemoglobin, platelets, haematocrit) | N95 and surgical masks had no detrimental effects on hemodynamic and haematologic function in both males and females. |
| Driver (2021) et al [11] | N = 31 recreationally active participants  (17 males, 14 females) | Incremental CPET using a Bruce treadmill protocol | K5 COSMED masks with and without cloth face masks | Exercise time, maximal oxygen consumption (VO2 max), VO2/kg, Respiratory exchange ratio (RER), minute ventilation (VE)/CO2 production (VCO2), VE, breathing reserve (BR), VO2/HR, respiratory frequency (RF) and tidal volume (TV), chronotropic index, max HR, RR, BP, SpO2, RPE  A scale of measuring subjective perceptions to mask wearing (humidity, heat, breathing resistance, itchiness, saltiness, feeling unfit, odour and fatigue) | Mask wearing impacted exercise performance (14% reduction in exercise time), physiological variables (VO2 max, VE, HR, RR and SpO2) and increased rating of perceived exertion. |
| Egger (2021) et al [13] | N = 16 well-trained male athletes (2 road cyclists, 8 mountain bikers, 6 triathletes) | Incremental test on a bicycle ergometer | Surgical mask  FFP2 mask  Loose spirometry mask was placed over the respective face masks and fixed and tightened with head straps | RPE, arterialized capillary blood from the hyperemised earlobe (blood lactate levels)  VO2, VCO2, respiratory minute ventilation (VE), HR, ECG, BP | Significant reduction of maximum performance, reduction in oxygen consumption, minute ventilation and reports of acute dyspnea was observed in both surgical mask and to a lesser extent, FFP2 mask. No difference in individual anaerobic threshold. |
| Epstein (2020) et al [19] | N = 16  (all males) | Standard cycle ergometry ramp protocol | Surgical mask  N95 respirator | HR, RR, BP, SpO2, time to exhaustion, EtCO2, RPE | No significant difference in HR, RR, BP, SpO2 and time to exhaustion. Exercising with N95 mask was associated with significant increase in EtCO2 levels, with the difference being more prominent as the load increased, reaching 8mmHg at exhaustion (none vs N95, P = 0.001). |
| Fikenzer (2020) et al [12] | N = 12  (all males) | Incremental exertion test on a semi-recumbent ergometer | Surgical mask  FFP2/N95 mask + spirometry mask was placed over the face masks and fixed with head straps in a leak-proof manner | BP, CO, SV, HR, arterio-venous oxygen difference (avDO2), cardiac work (CW)  FVC, FEV1, PEF, TIFF, VO2 max, VE  Capillary blood samples taken from earlobe (pH, pCO2, pO2, Lactate)  Quantification of comfort/discomfort of wearing a mask (humidity, heat, breathing resistance, itchiness, tightness, saltiness, feeling unfit, odor, fatigue and overall discomfort) | Pulmonary function parameters (FEV, PEF), ventilation and comfort of wearing masks were significantly affected in surgical and FFP2/N95 masks. |
| Freemas (2019) et al [23] | N = 14 recreationally trained males | Incremental exercise test to volitional exhaustion on cycle ergometry | Hans-Rudolph mouthpiece attached to a two-way non-rebreathing valve with a nose clip or while wearing a Hans-Rudolph 7450 V2 Series Face Mask | FVC, FEV1, FEV1/FVC, FEF  VE, VT, RR, %EFL, ERV, IRV, Peak insp flow, peak exp flow, duty cycle, VO2, VCO2, VE/VCO2, FeO2, FeCO2  HR  RPE and RPB | No significant differences in VE, breathing mechanics, gas exchange variables or RPB between the mouthpiece with nose clip and the face mask. |
| Lässing (2020) et al [15] | N = 14  (all males) | Double incremental exertion test (DIET)/lactate minimum test using cycle ergometer | Surgical face masks and Body plethysmography measurements with spirometry masks | FEV1, PEF, RAW airway resistance, VC  VO2, VCO2, VE, VE/VO2, VE/VCO2, VA (alveolar ventilation), VT (tidal volume), Ti (inspiratory time), Te (expiratory time), RR  SBP, DBP, CO, SV, HR, CW, avDO2, blood lactate concentration, RER, RPE, SO2%, Mean power output, exercise duration | Surgical mask wearing resulted in two-fold higher airway resistance, higher HR and CO. BP and rating of perceived exertion were similar in both no mask and mask wearing. |
| Roberge (2012) et al [18] | N = 20  (13 males, 7 females) | Exercise treadmill walking at a low-moderate work rate (5.6 km/h at 0 deg inclination) | Surgical mask | HR, RR, O2 saturation, SpO2, TcCO2, core and skin temperatures, mask dead space heat and relative humidity, skin temperature under masks, RPE, heat perceptions (RHP) | Surgical mask wearing resulted in mild increases in physiological responses (HR, RR, TcCO2). There was no clinically significant difference in subjective perceptions of exertion or heat. |
| Shaw (2020) et al [17] | N = 14  (7 males, 7 females) | Progressive Cycle ergometry exercise test to exhaustion | Surgical mask  Cloth mask | HR, blood oxygen saturation, RPE, tissue oxygenation index | Mask wearing had no effect on exercise performance (time to exhaustion, peak power), physiological variables (arterial oxygen saturation, tissue oxygenation index, HR) and RPE. |
| Umutlu (2021) et al [16] | N = 14 sedentary participants  (7 males, 7 females) | Incremental Bruce protocol on a treadmill | Surgical mask + CPET mask | HR, SBP, DBP  BF (breathing freq), RER (respi exchange ratio), EE (energy expenditure), VE, VO2, VCO2 | Surgical mask wearing resulted in lower VO2, VCO2 and VE (p < 0.001) and higher HR, SBP and DBP (p < 0.001). |
